# BBA, a Synthetic Derivative of 23-hydroxybutulinic Acid, Reverses Multidrug Resistance by Inhibiting the Efflux Activity of MRP7 (ABCC10)

**DOI:** 10.1371/journal.pone.0074573

**Published:** 2013-09-17

**Authors:** Jun-Jiang Chen, Atish Patel, Kamlesh Sodani, Zhi-Jie Xiao, Amit K. Tiwari, Dong-Mei Zhang, Ying-Jie Li, Dong-Hua Yang, Wen-Cai Ye, Si-Dong Chen, Zhe-Sheng Chen

**Affiliations:** 1 Guangdong Key Laboratory for Molecular Epidemiology, School of Public Health, Guangdong Pharmaceutical University, Guangzhou, China; 2 Department of Pharmaceutical Sciences, College of Pharmacy and Health Sciences, St. John’s University, Queens, New York, United States of America; 3 College of Pharmacy, Jinan University, Guangzhou, China; 4 Biosample Repository, Fox Chase Cancer Center, Philadelphia, Pennsylvania, United States of America; Enzo Life Sciences, Inc., United States of America

## Abstract

Natural products are frequently used for adjuvant chemotherapy in cancer treatment. 23-*O*-(1,4'-bipiperidine-1-carbonyl) betulinic acid (BBA) is a synthetic derivative of 23-hydroxybutulinic acid (23-HBA), which is a natural pentacyclic triterpene and the major active constituent of the root of *Pulsatilla* *chinensis* . We previously reported that BBA could reverse P-glycoprotein (P-gp/ABCB1)-mediated multidrug resistance (MDR). In the present study, we investigated whether BBA has the potential to reverse multidrug resistance protein 7 (MRP7/ABCC10)-mediated MDR. We found that BBA concentration-dependently enhanced the sensitivity of *MRP7*-transfected HEK293 cells to paclitaxel, docetaxel and vinblastine. Accumulation and efflux experiments demonstrated that BBA increased the intracellular accumulation of [^3^H]-paclitaxel by inhibiting the efflux of [^3^H]-paclitaxel from HEK293/MRP7 cells. In addition, immunoblotting and immunofluorescence analyses indicated no significant alteration of MRP7 protein expression and localization in plasma membranes after treatment with BBA. These results demonstrate that BBA reverses MRP7-mediated MDR through blocking the drug efflux function of MRP7 without affecting the intracellular ATP levels. Our findings suggest that BBA has the potential to be used in combination with conventional chemotherapeutic agents to augment the response to chemotherapy.

## Introduction

Chemotherapy is generally used for the treatment of various types of cancers. Many diverse chemotherapeutic agents can be used to control the growth, multiplication and spread of cancer cells. However, resistance to chemotherapeutic drugs is a significant factor that limits the potency of chemotherapy and causes failure of cancer treatment [1]. Simultaneous resistance to a number of structurally and functionally unrelated chemotherapeutic agents is the phenomenon known as multidrug resistance (MDR) [1,2]. One of the most important mechanisms responsible for MDR phenotype is the active efflux of the anticancer drugs from cancer cells by members of the ATP-binding cassette (ABC) transporter superfamily [3,4]. There are 49 genes in the human genome that encode ABC transporters. These transporters have been identified and grouped into seven subfamilies from A to G based on genome sequence similarities [5,6]. ABCB1 (also known as P-glycoprotein/P-gp), ABCC (also known as multidrug resistance protein/MRP) subfamily and ABCG2 (also known as breast cancer resistance protein/BCRP) are considered major players in the development of MDR in cancer cells [4,7]. The MRP subfamily of ABC transporters consists of nine members (MRP1-MRP9) and these nine MRP members involved in MDR represent the major share of the 12 members of the C subfamily of ABC transporters [8]. ABCC10 (also known as multidrug resistance protein 7/MRP7) is one distinct player of MRP subfamily in the development of MDR in cancer cells [9]. Recent studies have shown that MRP7 exhibits functional similarity to other drug efflux pumps of ABC transporters [9,10]. P-gp is the first discovered human ABC drug transporter, and transports a wide variety of hydrophobic compounds, including some of the most common anticancer drugs, such as taxanes, anthracyclines, vinca alkaloids and tyrosine kinase inhibitors (TKIs) [11]. MRP7 is a hydorphobic anion transporter that has the ability to confer resistance to certain natural agents, including taxanes and vinca alkaloids, that are also the substrates of P-gp [12]. In addition to natural agents, MRP7 is capable of conferring resistance to antiviral agents, such as tenofovir [13], and nucleoside-based agents gemcitabine and cytaratine (Ara-C) [14]. Moreover, the *Mrp7* ^*-/-*^ mouse has shown that Mrp7 contributes to the intrinsic resistance of cells and tissues to several chemotherapeutic agents including taxanes and Ara-C [15].

Reversing ABC transporter-mediated MDR can be achieved directly by developing inhibitors to block the drug efflux function of transporters and/or to regulate the expression of these pumps [16]. A great number of classic inhibitors from the first to the third generation have been discovered or synthesized to overcome ABC transporter-mediated MDR in the past three decades. However, the approach using inhibitors as chemosensitizers has had very little success in clinical studies [17]. The reasons behind unsuccessful clinical trials can be attributed to multiple factors, such as nonspecific toxicity of MDR inhibitors, the side effects of drug-drug interaction, toxic pharmacokinetic issues, and so on [3]. In recent years, several groups have reported single nucleotide polymorphisms (SNPs) in ABC transporters or in metabolic enzymes as other crucial factors for poor outcome of MDR inhibitors [18-20]. Selection of patients with different expression levels of these transporters in tumor tissue should be an important aspect to evaluate these inhibitors in clinical trials. Therefore, the use of inhibitors to reverse ABC transporters mediated-MDR is still a viable strategy for re-sensitizing MDR cancer cells to chemotherapeutic agents.

Natural products in combination of chemotherapeutic agents have shown good efficacy and low toxicity in clinical cancer therapy in history [21]. Search on active ingredients of natural products used for cancer treatment in clinic is one of the promising pathways to investigate novel inhibitors for reversal of ABC transporters-mediated MDR. Recently, our group reported 23-*O*-(1,4'-bipiperidine-1-carbonyl) betulinic acid (BBA), a synthetic derivative of 23-hydroxybutulinic acid (23-HBA), the major active constituent isolated from the root of *Pulsatilla* *chinensis* , could significantly reverse P-gp-mediated MDR [22,23]. The present study was designed to determine whether BBA could modulate MRP7-mediated MDR.

## Materials and Methods

### Materials

**Figure 1:**
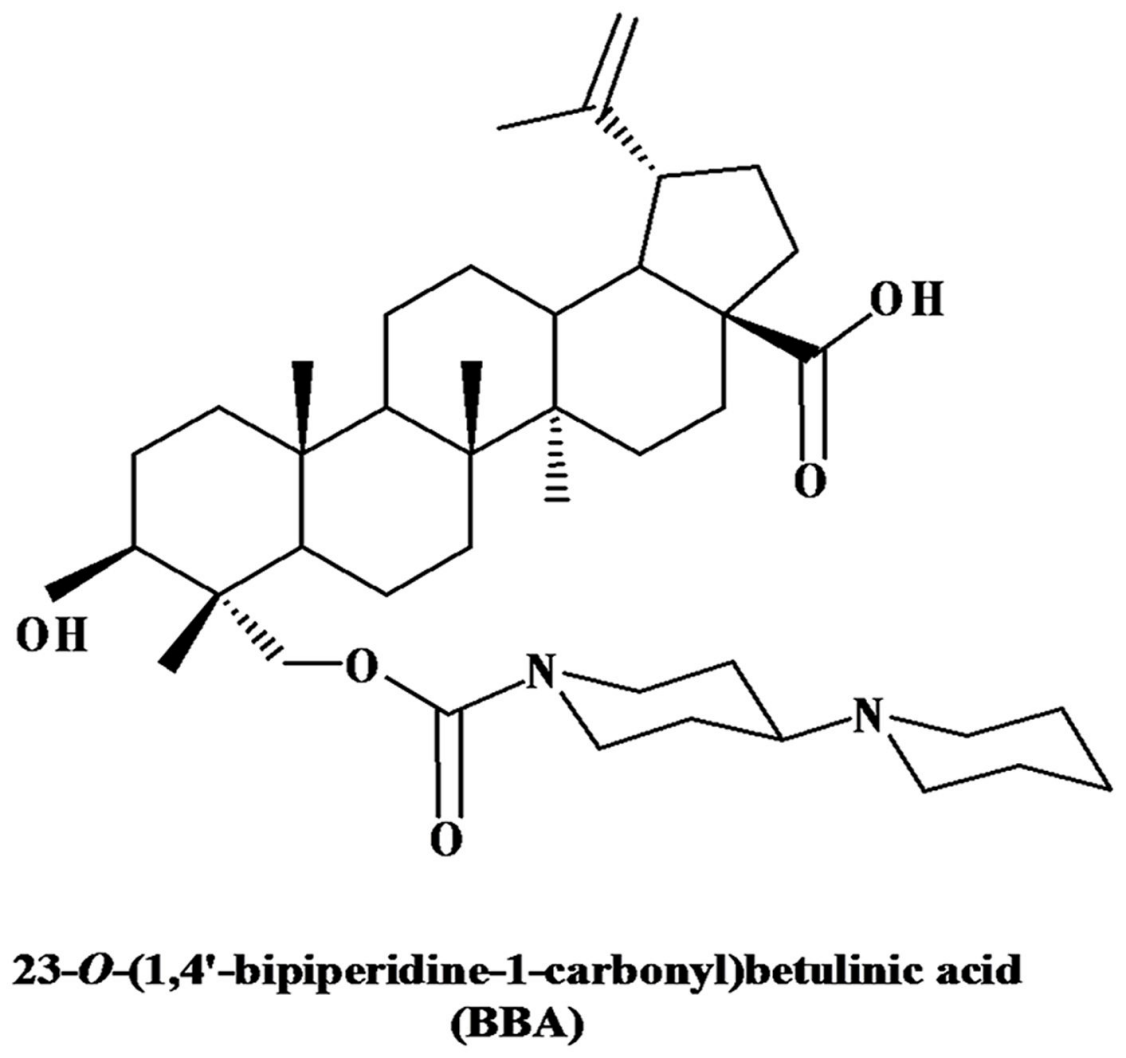
Chemical structure of BBA.

The BBA was synthesized, and isolated in the powder form using chromatography with a purity of > 98%, and solved in dimethyl sulfoxide (DMSO) as described previously [24]. The chemical structure of BBA is shown in Figure 1 Cepharanthine was generously provided by Daiichi Sankyo Pharmaceutical Co. Ltd (Tokyo, Japan). DMEM, trypsin 0.25%, FBS and penicillin/streptomycin were products of Hyclone (Logan, UT). [^3^H]-paclitaxel (46.5 Ci/mmol) was purchased from Moravek Biochemicals (Brea, CA). Monoclonal antibody C-219 (against P-gp) was obtained from Signet Laboratories Inc. (Dedham, MA), and the polyclonal antibody D-19 (against MRP7) was obtained from Santa Cruz Biotechnology (Santa Cruz, CA). Monoclonal antibody 14C10 (against GAPDH) was acquired from Cell Signaling Technology (Danvers, MA). Alexa flour 488 donkey anti-goat secondary antibody for immunofluorescence was purchased from Molecular Probes (Eugene, OR). The Titer-Glo Luminescent cell viability assay kit was purchased from Promega (Madison, WI). Docetaxel, paclitaxel, vinorelbine, vinblastine, vincristine, cisplatin, DMSO, MTT and other chemicals were purchased from Sigma Chemicals (St. Louis, MO).

### Cell lines and cell culture

HEK293/pcDNA3.1 and HEK293/MRP7 cells were established by transfecting HEK293 cells with either empty pcDNA3.1 vector or *MRP7* expressing vector as previously described [10]. The *MRP7* cDNA was generously provided by Dr. Gary Kruh (University of Illinois, Chicago, IL) and inserted into the pcDNA3.1 expression vector. Individual colonies were selected in medium containing G418 (1 mg/mL) and cultured for further analysis. The NCI-H23 cells cells were purchased from ATCC (Manassas, VA, USA). HEK293 cells transfected with ABCB1 were generously provided by Dr. Suresh V. Ambudkar (NCI, NIH, MD). All the cell lines were grown as adherent monolayer in flasks with DMEM supplemented with 10% FBS, 100 units/mL penicillin and 100 units/mL streptomycin under standard culture condition (37 °C, 5% CO_2_) in a humidified incubator.

### Cytotoxicity assay

MTT colorimetric assay was performed to analyze the cytotoxicity of BBA and the reversal effect of BBA on the sensitivity of anticancer drugs as previously described [25]. Briefly, The NCI-H23, HEK293/pcDNA3.1 and HEK293/MRP7 cells were seeded in 96-well plates in triplicates at 6000 cells/well in DMEM supplemented with 10% bovine serum at 37 °C for 24 h. For the cytotoxicity of BBA, various concentrations of BBA diluted with medium were added into the wells. For the reversal effect of BBA in MRP7-overexpressing cells, two sets of experiments were conducted. For the first set, three different non-toxic concentrations of BBA (1.25, 2.5 and 5 µM) were added into plates 1 h prior to the addition of the substrates of MRP7 (docetaxel, paclitaxel, vinorelbine, vinblastine and vincristine). For the second set of experiments, cells were pretreated with BBA at 5 µM for 1 h, medium was removed and cells were washed with PBS, and then medium-containing paclitaxel at different concentration was added into each well. These cells were incubated for 68 h, later 20 µL MTT solution (4 mg/mL) was added into each well. The plates were further incubated for 4 h, the medium was then discarded, and 100 µL of DMSO was added into each well to dissolve the formazan crystals then formed. The absorbance was determined at 570 nm by an OPSYS Microplate Reader from DYNEX Technologies (Chantilly, VA, USA). The degree of resistance was calculated by dividing the IC_50_ values (concentrations required to inhibit growth by 50%) for the HEK293/MRP7 cells by those of the parental HEK293/pcDNA3.1 cells. The resistance for NCI-H23 was calculated by dividing the IC_50_ obtained in the presence of paclitaxel by the IC_50_ obtained in the presence of each of the inhibitors cepharanthine or BBA. The Bliss method was used to calculate the IC_50_ values according to survival curves [26].

### [^3^H]-paclitaxel accumulation and efflux assay

The effect of BBA on the intracellular accumulation of paclitaxel in HEK293/pcDNA3.1 and HEK293/MRP7 cells was measured using [^3^H]-paclitaxel as previously described [27,28]. HEK293/pcDNA3.1 and HEK293/MRP7 cells were trypsinized and four aliquots from each cell line were suspended in the medium. Aliquots were pre-incubated with medium-only (control), BBA (2.5 and 5 µM) and cepharanthine (2.5 µM) at 37 °C for 2 h, and then incubated with 0.1 µM [^3^H]-paclitaxel for another 2 h. For efflux study, the cells were treated the same as drug accumulation study, and then washed three times with ice-cold PBS, suspended in fresh medium with or without BBA. Aliquots were evenly collected at various time points (0, 30, 60, 120 min). Samples from both accumulation and efflux experiments were washed by ice-cold PBS thrice and placed in scintillation fluid and radioactivity was measured in a Packard TRI-CARB 1900CA liquid scintillation analyzer from Packard Instrument Company (Downers Grove, IL).

### Preparation of total cell lysates and immunoblotting analysis

Due to the substrate similarity between MRP7 and ABCB1 the HEK293/pcDNA3.1 cells and HEK293/MRP7 cells were analyzed for the presence of any traces of ABCB1 in cells used for the present study. The cells once confluent were collected and rinsed twice with ice-cold PBS and the total cell lysates were collected and maintained in RIPA (Radioimmunoprecipitation assay) buffer (Sigma Chemicals) (1 × PBS, 1% Nonidet P-40, 0.5% sodium deoxycholate, 0.1% SDS, 100 µg/ml phenylmethylsulfonyl fluoride, 10 µg/ml aprotinin, 10 µg/ml leupeptin) for 30 min with occasional rocking followed by centrifugation at 13,000 × g at 4 °C for 15 min. The protein concentration was determined by bicinchoninic acid-based protein assay (Thermo Scientific, Rockford, IL). Equal amounts of total cell lysates (40 µg of protein) were resolved by 4-12% SDS-PAGE and electrophoretically transferred onto PVDF membranes. After incubating in blocking solution containing 5% skim milk in TBST buffer (10 mM Tris-HCL, PH 8.0, 150 mM NaCl and 0.1% Tween 20) at room temperature for 1 h, the membranes were immunoblotted overnight with primary antibodies, anti-P-gp (1:200 dilution) anti-MRP7 (1:200 dilution) and anti-GAPDH (1:1000 dilution) at 4 °C for overnight. Subsequently, the membranes were washed three times for 15 min with TBST buffer and incubated at room temperature for 2 h with HRP-conjugated secondary antibody (1:2000 dilution). The protein-antibody complex was detected using the enhanced Phototope TM-HRP Detection Kit (Cell Signaling Technology) and exposed to Kodak medical X-ray processor (Kodak, Rochester, NY) [25]. Later to determine the effect of BBA on the expression of MRP7, HEK293/MRP7 cells were incubated with 5 µM BBA for different time periods (0, 24, 48 and 72 h). Then the cells were harvested and rinsed twice with ice-cold PBS. Total cell lysates were collected and the protein was resolved and detected as described earlier.

### Immunofluorescence analysis

HEK293/MRP7 cells (1 × 10^4^) were seeded in 24-well plates and cultured overnight. BBA at 5 µM was added into the wells at different time periods (0, 24, 48 and 72 h) and then cultured at 37 °C in a humidified incubator containing 5% CO_2_. Cells were washed with PBS and fixed with 4% paraformaldehyde for 15 min at room temperature and then rinsed with PBS three times. Non-specific reaction was blocked with 1% BSA for 1 h at room temperature. A polyclonal antibody D-19 against MRP7 (1:200) was added and incubated overnight. Then, cells were incubated with Alexa Flour 488 donkey anti-goat IgG (1:2000) for 1 h at room temperature. DAPI was used for the nuclear staining. Immunofluorescent images were taken using an inverted microscope (model IX70; Olympus, Center Valley, PA) with IX-FLA fluorescence and a CCD camera [29].

### Intracellular ATP level measurement

Briefly, HEK293/MRP7 cells were seeded in Lumitrac 96 well plate from Griener Bio one (Monroe, NC) at 6000 cells per well in culture medium. After overnight incubation, various concentrations of BBA (1.25, 2.5 and 5 µM) was added and further incubated for another 72 h. Equal volume of Cell Titer-Glo reagent was added for 10 min at room temperature and cellular ATP level was measured by a microplate reader (DTX880, Beckman)

### Statistical analysis

All experiments were repeated at least three times and the differences were determined using Student’s *t*-test. The statistical significance was determined at *P* < 0.05.

## Results

### Effect of BBA on the sensitivity of anticancer drugs in HEK293/pcDNA3.1 and HEK293/MRP7 cells

**Figure 2:**
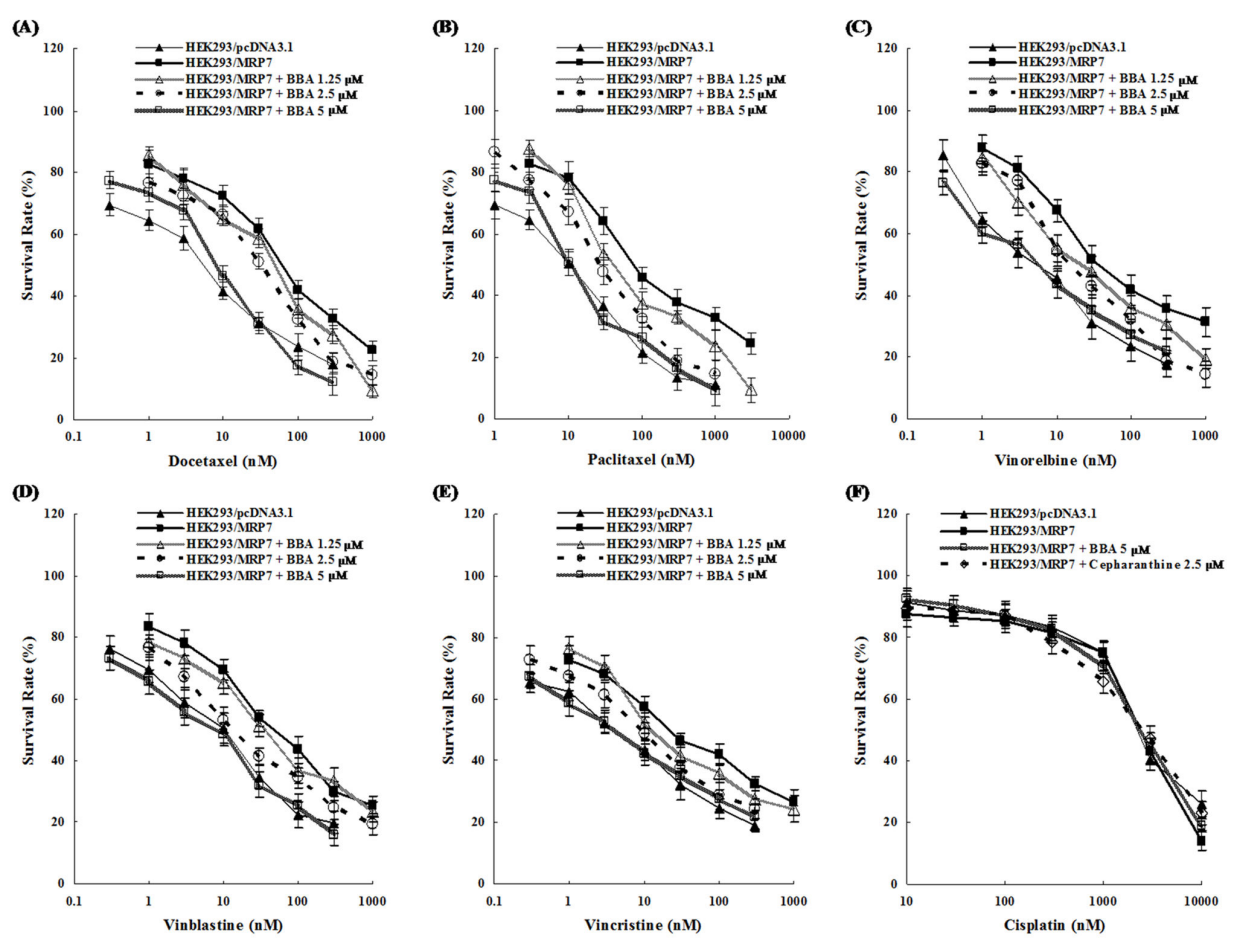
BBA increases the cytotoxicity of anticancer drugs in concentration-dependent manner. Panel A, B, C, D, E, F: the survival curves of parental HEK293/pcDNA3.1 and HEK293/MRP7 cells without reversal agent and HEK293/MRP7 cells in the presence of cepharanthine (a known inhibitor of MRP7) at 2.5 µM or BBA at 1.25, 2.5 and 5 µM at the different concentrations of docetaxel, paclitaxel, vinorelbine, vinblastine, vincristine and cisplatin (a non-substrate of MRP7), respectively. Cell survival was determined by MTT assay as described in “Materials and Methods”. Data are means ± SD of at least three independent experiments performed in triplicate.

Prior to analyzing the reversal efficacy of BBA, we tested its cytotoxic effect in HEK293/pcDNA3.1 and HEK293/MRP7 cells using MTT assay. The results showed that BBA had no significant toxicity on both HEK293/pcDNA3.1 and HEK293/MRP7 at concentrations up to 30 µM (Figure S1). We then investigated the cytotoxicity of anticancer drugs (docetaxel, paclitaxel, vinorelbine, vinblastine, vincristine or cisplatin) alone and in combination with BBA at non-toxic concentrations (1.25, 2.5 and 5 µM) in the HEK293/pcDNA3.1 and HEK293/MRP7 cells. As shown in Table 1 and Figure 2, HEK293/MRP7 cells in comparison to parental HEK293/pcDNA3.1 cells, exhibited a significant resistance to various MRP7 substrate anticancer drugs, such as docetaxel, paclitaxel, vinorelbine, vinblastine and vincristine, which is consistent with our previous reports [12,30]. BBA concentration-dependently increased the cytotoxicity of above-mentioned MRP7 substrates in HEK293/MRP7 cells. Cepharanthine, the known MRP7 inhibitor, as a positive control at 2.5 µM, completely reversed the resistance of HEK293/MRP7 cells to docetaxel, paclitaxel, vinorelbine, vinblastine and vincristine. In contrast, BBA and cepharanthine both did not significantly enhance the sensitivity of HEK293/MRP7 cells to cisplatin, a non-substrate of MRP7. In the parental HEK293/pcDNA3.1 cells, there was no significant difference between the IC_50_ values of docetaxel, paclitaxel, vinorelbine, vinblastine and vincristine in the presence or absence of BBA (*P* > 0.05, Table 1). The survival rates of HEK293/MRP7 cells decreased significantly after co-incubation of anticancer drugs with BBA at 2.5 and 5 µM (Figure 2). The pretreatment with BBA for 1 h did not reverse the paclitaxel resistance in HEK/MRP7 cells, which suggest that the effect of BBA on MRP7 is reversible (data not shown).

**Table 1:** The effect of BBA on the sensitivity of HEK293/pcDNA3.1 and HEK293/MRP7 cells to docetaxel, paclitaxel, vinorelbine, vinblastine, vincristine and cisplatin.

Compounds	IC_50_ ± SD^a^ (nM)	
	HEK293/pcDNA3.1	HEK293/MRP7
Docetaxel	6.57 ± 0.45 (1.0)^b^	71.46 ± 4.91 (10.9)
+B2B1 1.25 µM	6.22 ± 0.78 (0.95)	56.85 ± 6.83* (8.65)
+B2B1 2.5 µM	5.95 ± 0.41 (0.91)	34.39 ± 4.28** (5.23)
+B2B1 5 µM	5.63 ± 0.56 (0.86)	8.88 ± 0.79** (1.35)
+Cepharanthine 2.5 µM	4.98 ± 0.32** (0.76)	8.81 ± 0.94** (1.34)
Paclitaxel	10.38 ± 0.87 (1.0)	84.18 ± 7.38 (8.11)
+B2B1 1.25 µM	10.11 ± 0.62 (0.97)	44.57 ± 4.19** (4.29)
+B2B1 2.5 µM	9.35 ± 0.99 (0.90)	27.68 ± 2.17** (2.67)
+B2B1 5 µM	9.21 ± 0.74 (0.89)	10.60 ± 0.88** (1.02)
+Cepharanthine 2.5 µM	7.89 ± 0.93** (0.76)	8.48 ± 0.94** (0.82)
Vinorelbine	6.29 ± 0.62 (1.0)	42.36 ± 3.16 (6.73)
+B2B1 1.25 µM	6.63 ± 0.57 (1.05)	24.58 ± 2.39** (3.91)
+B2B1 2.5 µM	6.37 ± 0.67 (1.01)	17.64 ± 1.48** (2.8)
+B2B1 5 µM	6.21 ± 0.48 (0.99)	6.58 ± 0.77** (1.05)
+Cepharanthine 2.5 µM	5.82 ± 0.49 (0.93)	5.93 ± 0.64** (0.94)
Vinblastine	10.73 ± 1.14 (1.0)	55.53 ± 6.2 (5.18)
+B2B1 1.25 µM	9.53 ± 0.83 (0.89)	36.69 ± 2.79** (3.42)
+B2B1 2.5 µM	9.25 ± 0.74 (0.86)	15.31 ± 1.38** (1.43)
+B2B1 5 µM	8.85 ± 0.93 (0.82)	8.66 ± 0.96** (0.81)
+Cepharanthine 2.5 µM	8.72 ± 1.02 (0.81)	8.49 ± 0.87** (0.79)
Vincristine	4.83 ± 0.56 (1.0)	23.63 ± 2.04 (4.89)
+B2B1 1.25 µM	4.75 ± 0.48 (0.98)	14.13 ± 1.57** (2.93)
+B2B1 2.5 µM	4.98 ± 0.61 (1.03)	9.29 ± 1.06** (1.92)
+B2B1 5 µM	4.56 ± 0.44 (0.94)	4.87 ± 0.58** (1.01)
+Cepharanthine 2.5 µM	4.52 ± 0.32 (0.94)	4.74 ± 0.46** (0.98)
Cisplatin	2443.40 ± 146.23 (1.0)	2562.44 ± 122.48 (1.05)
+B2B1 5 µM	2394.17 ± 160.83 (0.98)	2572.88 ± 131.27 (1.05)
+Cepharanthine 2.5 µM	2614.76 ± 169.26 (1.07)	2679.91 ± 153.79 (1.10)

* *P* < 0.05; **P < 0.01. ^a^ IC_50_: concentration that inhibited cell survival by 50%. Data are means ± SD of at least three independent experiments performed in triplicate. ^b^ Fold-resistance was calculated as the IC_50_ values of anticancer drug for HEK293/pcDNA3.1 and HEK293/MRP7 cells with or without reversal agent divided by the IC_50_ values of respective anticancer drug for HEK293/pcDNA3.1 cells without reversal agent.

### Effect of BBA on the sensitivity of paclitaxel in NCI-H23 cells

**Figure 3:**
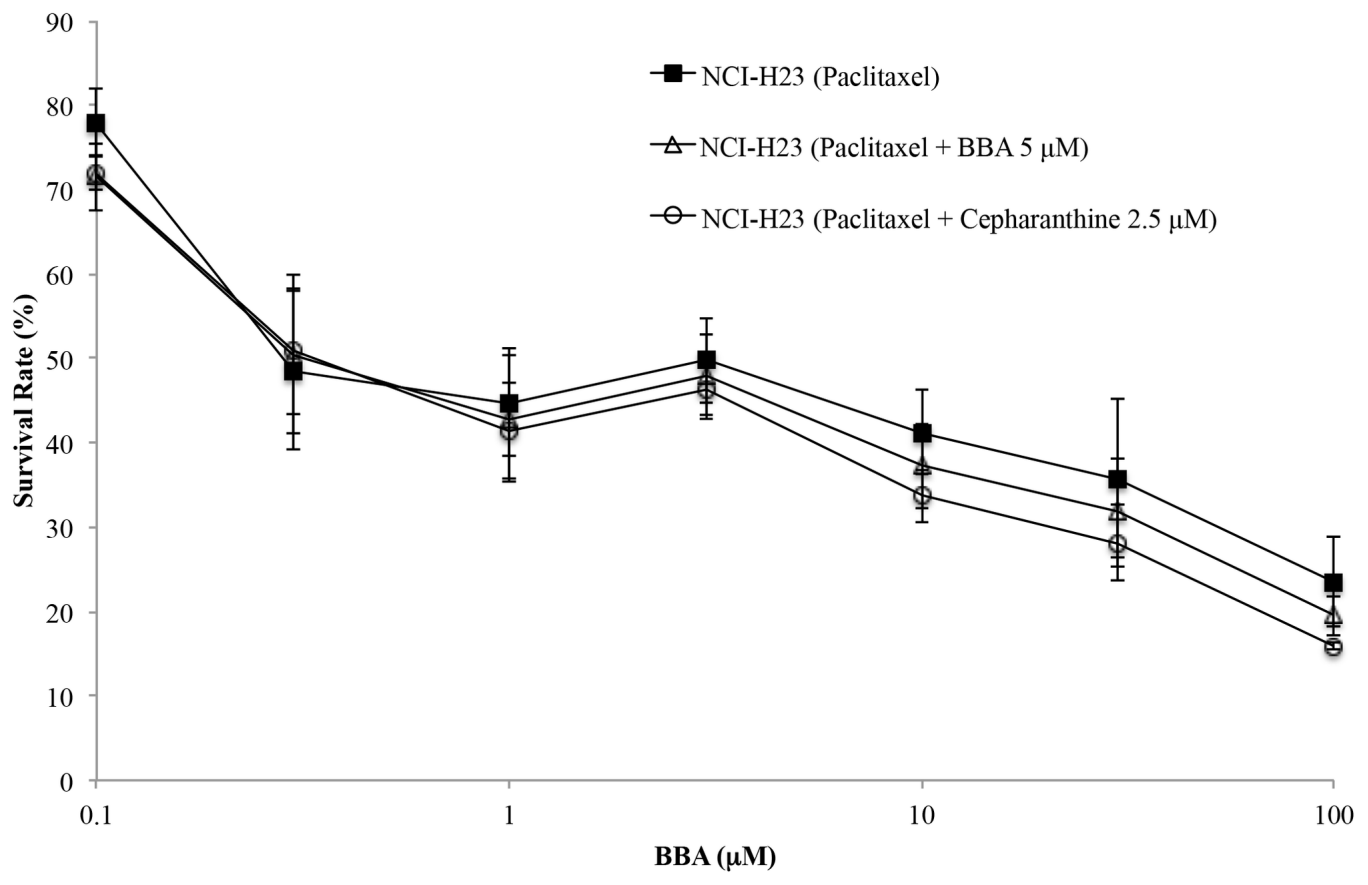
BBA does not affect the sensitivity to paclitaxel in NCI-H23 cells.

Some reports have shown the *MRP7* gene expression in NCI-H23, a non-small cell lung cancer cell line. Therefore, we performed cell cytotoxicity assays to determine the ability of BBA at 5 µM to reverse MRP7-mediated drug resistance to paclitaxel in this cell line [31]. There was no change in the resistance folds either in the presence or absence of BBA at 5 µM or cepharanthine at 2.5 µM concentration (Table 2 and Figure 3).

**Table 2:** The effect of BBA on the sensitivity of NCI-H23 cells to paclitaxel.

Compounds	IC_50_ ± SD^a^ (nM)
	NCI-H23
Paclitaxel	0.378 ± 0.004 (1.0)^b^
+B2B1 5 µM	0.316 ± 0.001 (0.837)
+Cepharanthine 2.5 µM	0.391 ± 0.009 (1.35)

^a^ IC_50_: concentration that inhibited cell survival by 50%. Data are means ± SD of at least three independent experiments performed in triplicate.

^b^ Fold-resistance was calculated by dividing the IC_50_ obtained in the presence of paclitaxel by the IC_50_ obtained in the presence of each of the inhibitors cepharanthine or BBA.

 The survival curves of NCI-H23 cells in the presence and absence of BBA (5 µM) and cepharanthine (2.5 µM) at different concentration of paclitaxel. Cell survival was determined by MTT assay as described in “Materials and Methods”. Data are means ± SD of at least three independent experiments performed in triplicate.

### Effect of BBA on the intracellular accumulation of [^3^H]-paclitaxel in HEK293/MRP7 cells

**Figure 4:**
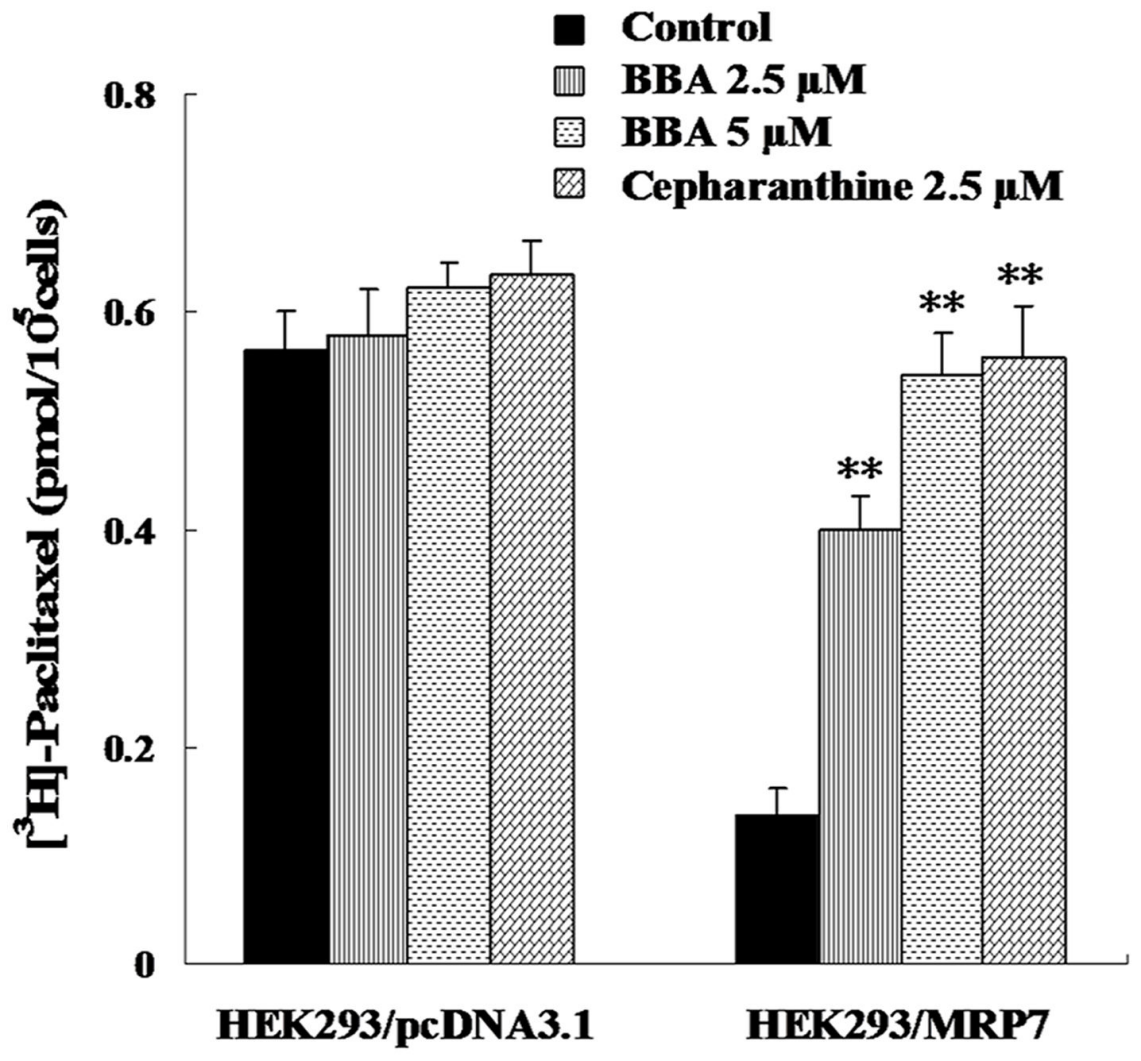
BBA increases the intracellular accumulation of [^3^H]-paclitaxel in HEK293/MRP7 cells. The intracellular accumulation of [^3^H]-paclitaxel was measured by scintillation counting after cells were pre-incubated with or without BBA or cepharanthine (a known inhibitor of MRP7) for 2 h at 37 °C and then incubated with 0.1 µM [^3^H]-paclitaxel for another 2 h at 37 °C. Data points represent the means ± SD of triplicate determinations. Experiments were performed at least three independent times. ** *P* < 0.01, for values versus those in the control group.

To confirm the effect of BBA on the drug accumulation inside the cells, the intracellular accumulation of [^3^H]-paclitaxel study was determined. The intracellular concentration of [^3^H]-paclitaxel in HEK293/MRP7 cells was significantly lower (24.3%) than that in parental HEK293/pcDNA3.1 cells (100%) as shown in Figure 4. However, after the cells were incubated with BBA at 2.5 or 5 µM for 2 h, the intracellular accumulation of [^3^H]-paclitaxel in HEK293/MRP7 cells was significantly increased by 2.9- and 4.0-fold, when compared to 2.5 µM of cepharanthine as a positive control by 4.1-fold. Neither BBA nor cepharanthine significantly affected the intracellular levels of [^3^H]-paclitaxel in HEK293/pcDNA3.1 cells (Figure 4).

### Effect of BBA on the efflux of [^3^H]-paclitaxel in HEK293/MRP7 cells

**Figure 5:**
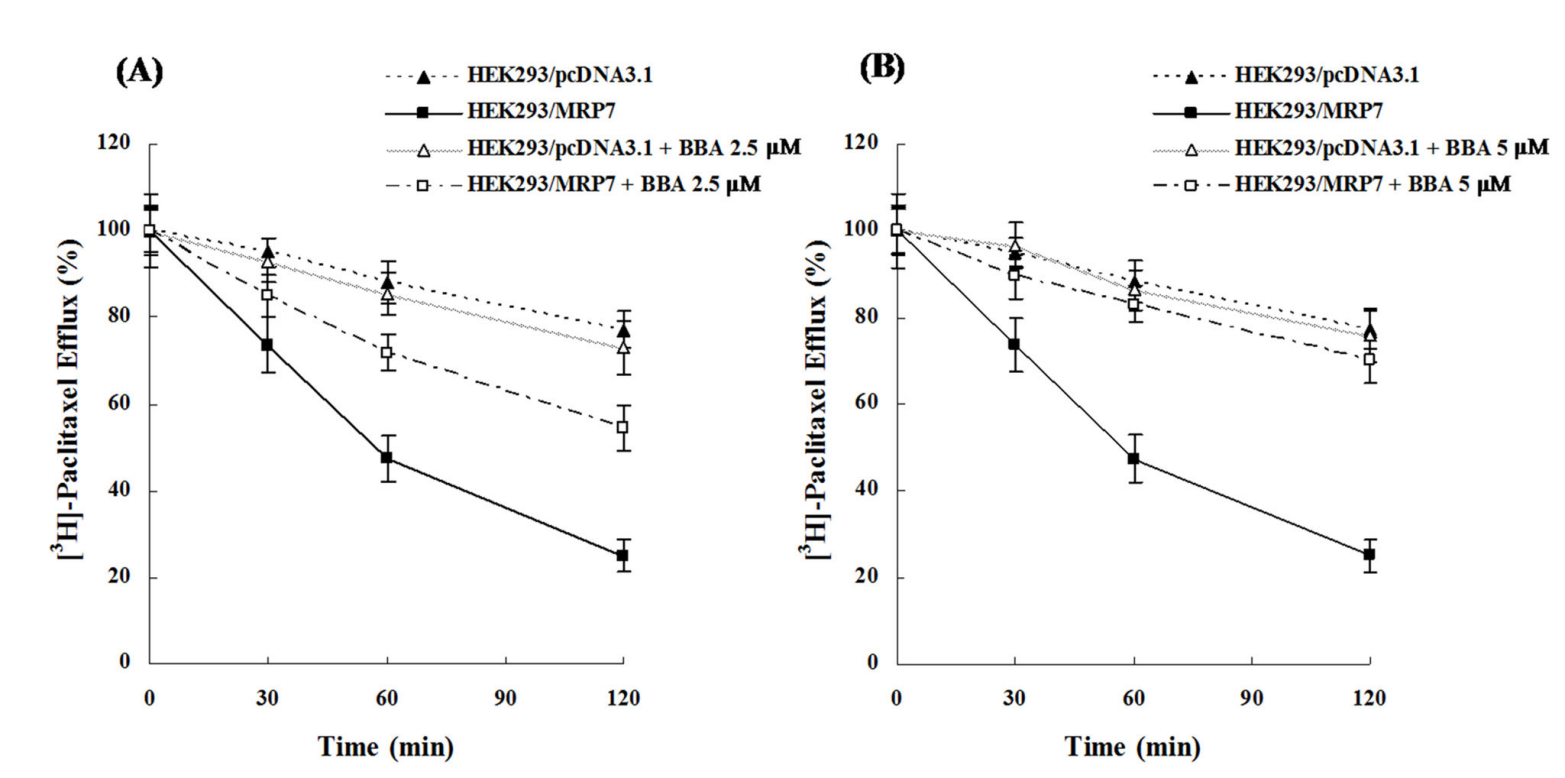
BBA inhibits the efflux of [^3^H]-paclitaxel mediated by MRP7 in HEK293/MRP7 cells. Cells were pre-incubated with or without BBA at 2.5 µM (A) or 5 µM (B) for 2 h at 37 °C and further incubated with 0.1 µM [^3^H]-paclitaxel for another 2 h at 37 °C. Cells were then incubated in the fresh medium with or without BBA at 2.5 or 5 µM for different time periods at 37 °C. Thereafter cells were collected and the intracellular levels of [^3^H]-paclitaxel were measured by scintillation counting. A time course versus percentage of intracellular [^3^H]-paclitaxel was plotted (0, 30, 60 and 120 min). Data points represent the means ± SD of triplicate determinations. Experiments were performed at least three independent times.

To ascertain whether the increase in the intracellular accumulation of [^3^H]-paclitaxel in the presence of BBA was due to the inhibition of [^3^H]-paclitaxel efflux by MRP7, we designed a time course study to measure intracellular [^3^H]-paclitaxel levels in the presence or absence of BBA. As shown in Figure 5, a higher percentage of intracellular [^3^H]-paclitaxel was extruded in HEK293/MRP7 cells than that in HEK293/pcDNA3.1 cells. However, in the presence of BBA at 2.5 or 5 µM, there was a significant decrease in the efflux of intracellular [^3^H]-paclitaxel at different time periods (0, 30, 60 and 120 min) from HEK293/MRP7 cells, but not from the parental HEK293/pcDNA3.1 cells. The intracellular accumulation of [^3^H]-paclitaxel at 0 min was set as 100%, and at 30, 60 and 120 min, the percentages of the accumulated [^3^H]-paclitaxel were 73.71%, 47.32% and 25.01%, respectively, of that remained in HEK293/MRP7 cells in the absence of BBA. When HEK293/MRP7 cells were incubated with BBA at 2.5 µM, the percentage of the intracellular [^3^H]-paclitaxel at 30, 60 and 120 min increased significantly to 84.99%, 71.93% and 54.28%, respectively (Figure 5A). BBA at 5 µM significantly increased the percentages of the intracellular [^3^H]-paclitaxel to 89.35%, 83.15% and 70.22% at 30, 60 and 120 min, respectively (Figure 5B). We thus concluded that BBA reverses MRP7-dependent efflux of paclitaxel

### Determining the expression levels of ABCB1 and MRP7 and effect of BBA on the expression of MRP7

**Figure 6:**
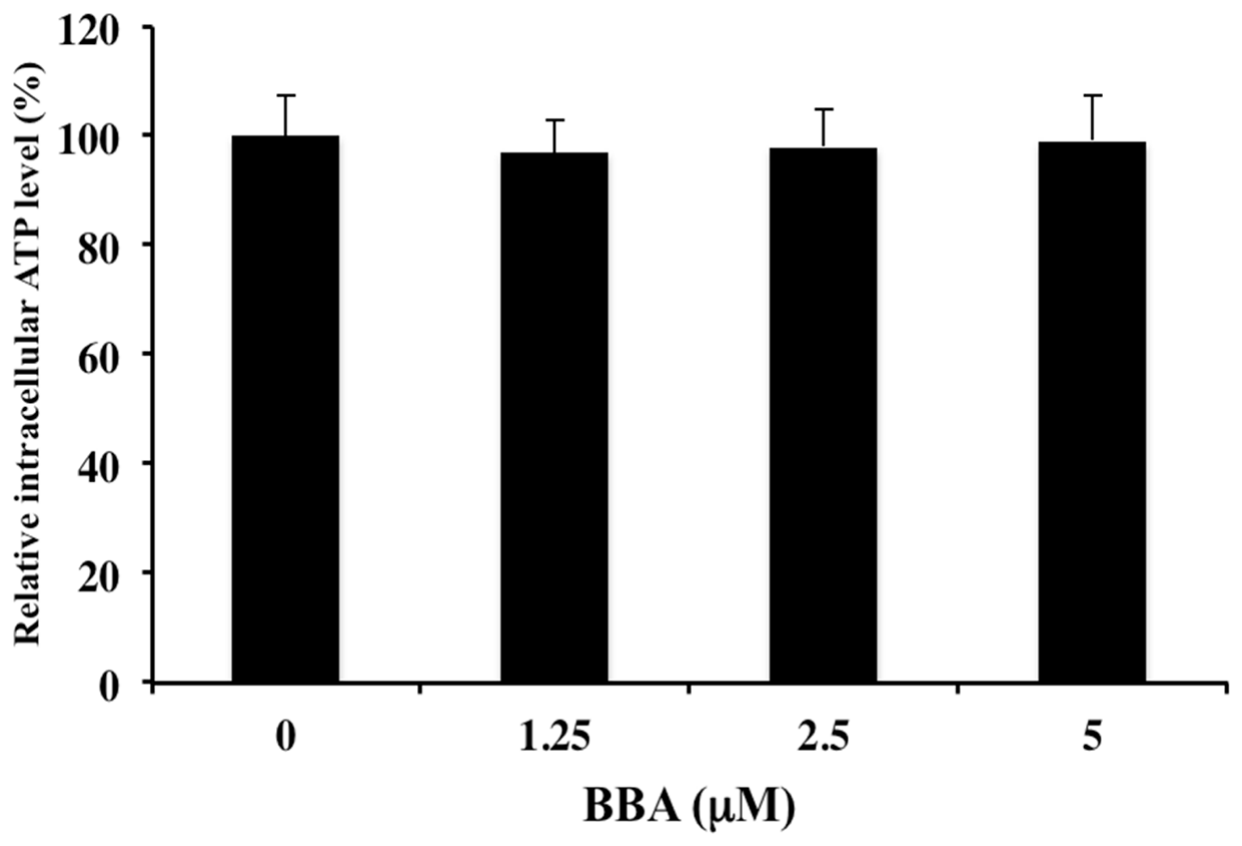
Immunoblotting of ABCB1/P-gp (A) and MRP7 (B) in HEK293 and HEK293/MRP7 cells and Immunofluorescence detection of MRP7 in HEK293/MRP7 cells (C). Cell lysates were prepared from HEK293/pcDNA3.1, HEK293/ABCB1 and HEK293/MRP7 cells (A), HEK293/MRP7 cells incubated with 5 µM BBA for different time periods (0, 24, 48 and 72 h) (B). Equal amounts (40 µg of protein) of total cell lysates were used for each sample. The localization of MRP7 by immunofluorescence was done on paraformaldehyde fixed cells using polyclonal antibody D19 against MRP7 and Alexa Flour^®^ 488 donkey anti-goat IgG(C). DAPI was used for nuclear counterstaining. Results from a representative experiment are shown. Similar results were obtained in two other trials.

To determine the expression of ABCB1, HEK293/pcDNA3.1 and HEK293/MPR7 cell lysates were treated with the ABCB1 antibody, the results obtained showed no traces of ABCB1 in either of the two cell lines used in the present study as shown in Figure 6A. Thereby confirming the resistance towards MRP7 substrates in HEK293/MRP7 cells to be solely due to MRP7. Further to evaluate the effect of BBA on the expression of MRP7, HEK293/MRP7 cells were treated with BBA at 5 µM for 0, 24, 48 and 72 h, and the expression levels of MRP7 were examined by immunoblotting analysis. The results shown in Figure 6B indicate that BBA did not significantly alter the protein expression levels of MRP7 in HEK293/MRP7 cells. We also analyzed the expression of MRP7 in NCI-H23 cell line. Surprisingly, no endogenous MRP7 was detected (data not shown).

### Effect of BBA on the localization of MRP7

The localization of MRP7 after BBA treatment was evaluated by immunofluorescence. As shown in Figure 6C, there was no alteration of MRP7 protein localization after the treatment with BBA at 5 µM for different time points (0, 24, 48 and 72 h). Both immunoblotting (Figure 6A) and immunocytochemical (Figure 6C) experiments suggested that BBA does not alter the expression and/or localization of the MRP7 transporter in HEK293/MRP7 cells providing evidence that reversal of MRP7-mediated MDR is due to the inhibition of drug efflux function of MRP7.

### Effect of BBA on the intracellular ATP level in HEK293/MRP7 cell line

**Figure 7:**
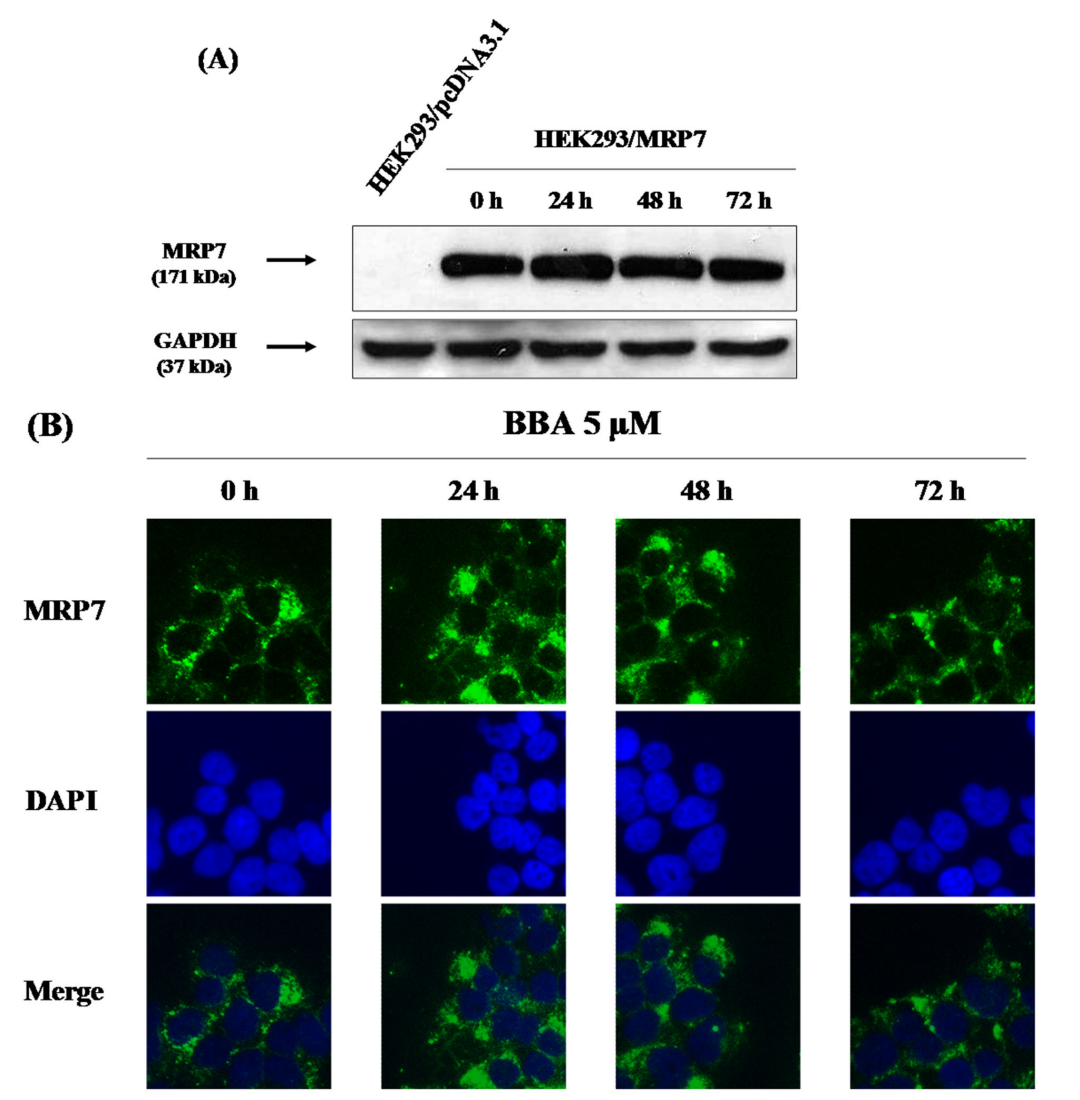
BBA does not affect the intracellular ATP levels in HEK293/MRP7 cells. HEK293/MRP7 cells were incubated with or without BBA at 1.25, 2.5 and 5 µM for 72 h. The intracellular ATP levels were measured as described in materials and methods. Data points represent the mean ± SD of triplicate determinations. Experiments were performed at least three independent times.

MRP7-mediated drug efflux function is dependent on energy obtained from ATP. BBA reverses MRP7-mediated MDR by inhibiting the ATP dependent efflux function of MRP7 in HEK293/MRP7 cell line. Thus, we analyzed the effect of BBA on intracellular ATP level in HEK293/MRP7 cells. We found that BBA up to 5 µM concentrations did not significantly affect the intracellular ATP level in HEK293/MRP7 cell line (Figure 7).

## Discussion

Natural products have been used for combating human ailments in history. In recent years, natural products were investigated and used as adjuvant chemotherapy for cancer treatment [21,32]. The root of *Pulsatilla* *chinensis* , a traditional Chinese herbal medicine, has been widely used in the treatment of malaria, bacterial infections and malignant tumor [33]. There was a previous report that 23-HBA, the main component of *Pulsatilla* *chinensis* , synergizes the cytotocxicity of doxorubicin both *in vitro* and *in vivo*. This indicted that 23-HBA has the potential to be developed as a novel MDR modulator [23]. These kinds of MDR modulators isolated from natural sources are named as the so-called “fourth generation” modulators and currently being actively explored as lead compounds for chemical modification [34-36]. Based on structural modification of 23-HBA as previously described, a large number of 23-HBA derivatives had been synthesized to improve the pharmacological property and bioactivity [24]. On the basis of our preliminary screening results, we found that BBA possesses the best MDR reversal activity among the 23-HBA derivatives [24]. Recently, we reported for the first time that BBA could potently reverse P-gp-mediated MDR by directly inhibiting the transport function of P-gp and increase the intracellular accumulation of chemotherapeutic agents in P-gp-overexpressing cells *in vitro*. In addition, BBA could also reverse P-gp-mediated resistance to paclitaxel in nude mouse xenograft model [22]. In the present study, we examined whether BBA could reverse MRP7-mediated drug resistance. We performed experiments using HEK293/pcDNA3.1 and HEK293/MRP7 transfected cell lines. The expression of MRP7 along with the absence of ABCB1/P-gp in HEK293/MRP7 cell line was detected and confirmed by an immunoblot analysis (Figure 6 A and B).

BBA at 5 µM was able to completely reverse the MDR mediated by MRP7, as evidenced with cytotoxicity assay data (Table 1, Figure 2). BBA potently sensitized MRP7-overexpressing cells to MRP7 substrates docetaxel, paclitaxel, vinorelbine, vinblastine and vincristine. The ability of BBA to sensitize HEK293/MRP7 cells to paclitaxel was reversible (data not shown). BBA did not sensitize the cells to cisplatin (a non-substrate of MRP7) and had no significant effect on the drug sensitivity of the parental HEK293/pcDNA3.1 cells. Recently, Oguri et al. reported endogenous gene expression of MRP7 in NCI-H23 cells, therefore, the ability of BBA at 5 µM to reverse MRP7-mediated drug resistance was evaluated using cell cytotoxicity assay in NCI-H23 cells [31]. Surprisingly, there was no significant change in the resistance to paclitaxel either in the presence or absence of BBA and cepharanthine (Table 2 and Figure 3). Then, we conducted a Western blot analysis to verify the expression level of MRP7 protein in NCI-H23 cells. The expression of MRP7 was not detected in cell lysates from NCI-H23 cells (data not shown). This result confirmed that the inability of BBA to enhance the cytotoxicity of paclitaxel in NCI-H23 and suggested that BBA reverses MDR in HEK293/MRP7 cells is associated with MRP7 transporter.

Consistent with the cytotoxicity results, the drug accumulation data indicated that BBA significantly enhanced the intracellular accumulation of paclitaxel in HEK293/MRP7 cells. Because MRP7 is a drug efflux pump that contributes to the decrease of intracellular paclitaxel concentrations, a time course efflux study was performed to further confirm the accumulation results. Indeed, the efflux study showed that the efflux of intracellular paclitaxel was significantly blocked by BBA in the HEK293/MRP7 cell lines in comparison to those treated without BBA. Hence, the accumulation and efflux data along with cytotoxicity results indicate that BBA is targeting the MRP7 transporter.

The reversal effect of MRP7-mediated MDR by BBA could be due to the inhibition of the drug efflux function of MRP7 or alteration of the expression of MRP7. The immunoblotting and immunofluorescence analyses data demonstrate that no significant alterations in protein expression or localization of MRP7 from plasma membranes in HEK293/MRP7 cells in the presence of BBA at 5 µM for up to 72 h. These findings further indicate that BBA inhibits the drug efflux function of MRP7 rather than down-regulating the expression of MRP7.

ATP depletion has been shown to restore drug accumulation in resistant cells [37]. In has been suggested that agents inducing the sensitivity might be inhibiting the glycolytic pathway and reduce intracellular ATP levels in treated MDR cells, as these cells have increased metabolic requirement for ATP [38,39]. However, our results showed that BBA does not affect the intracellular ATP level in HEK293/MRP7 cells. The drug sensitivity study results (Figure S1) showed that there is no significant difference of cytotoxicity of BBA on both parental and resistant cell lines; this suggests that BBA may not be a substrate of MRP7. Most likely, BBA could directly bind to MRP7 like its direct interaction on P-gp. Previously, the [^125^I] iodoarylazidoprazosin (IAAP) photoaffinity labeling experiment and molecular docking model of binding BBA demonstrated the binding site of BBA to P-gp is partly coincident with the site of verapamil and IAAP [22]. Since MRP7 and P-gp both have similar characteristics on MDR and could transport identical chemotherapeutic drugs [11], molecular docking model of binding BBA to MRP7 is needed to further investigate the interaction between BBA and MRP7 in future study. Taken together with previous study that BBA is an inhibitor of the P-gp efflux pump and a reversal agent that partially reverses SN-38 resistance in the BCRP-overexpressing HEK293 cells, BBA might be effective in restoring the sensitivity of tumors to certain anticancer drugs by inhibiting the drug efflux activity of P-gp, MRP7 and/or BCRP.

MRP7 is a relative newly found ABC transporter, but it may play a role in the intrinsic sensitivity of tissues and tumors in which it is expressed. The transcript of *MRP7* has been reported in a wide range of normal tissues, including testis, skin, colon, spleen and stomach [40,41]. In addition, the transcript expression of *MRP7* was also found in several tumor specimens of the breast, lung, colon, prostate, ovary and pancreas [41]. Recently, Hlavata et al reported that the transcript level of *MRP7* in tumor tissues from colorectal cancer patients correlated with the tumor grade [42]. Although, our *MRP7*-transfected cells had only low level of resistance to paclitaxel compared to *ABCB1*-transfected cells [22,43], it had been reported that the gene expression levels of *MRP7* was much higher than that of *ABCB1* in both paclitaxel-resistance and vinorelbine-resistance NSCLC (nonsmall cell lung cancer) cell lines [31,44]. In addition, Naramoto et al. reported that induction of MRP7 and P-gp expression in vincristine-treated salivary gland adenocarcinoma cells [45]. It is not difficult to speculate that MRP7, similarly to P-gp, may act as an inducible transporter conferring resistance to certain anticancer drugs. Moreover, a recent report showed up-regulation of MRP7 in HCC (hepatocellular carcinoma) patient samples compared with adjacent healthy liver [46]. Based on the above results and previous report [43], MRP7 expression might be correlated to the treatment response in certain cancers in the context of treatment with taxanes. However, MRP7 is at early stages of investigation, further clinical investigation is required. Although, few MRP7 inhibitors have been identified, none have been tested in clinics yet. BBA as a dual inhibitor of P-gp and MRP7 is promising compared to other MDR inhibitors, like cepharanthine [22]. Consistent with our previous findings [47], the cytotoxicity assay revealed that cepharanthine, compared to BBA, increased the cytotoxicity of taxanes in HEK293/pcDNA3.1 cells (Table 1). There was no significant reduction of the body weight in nude mouse after treatment with BBA at effective reversal concentration of 15 mg/kg [22]. Although the plasma concentration of BBA was not determined in the previous nude mouse study, the plasma concentration of 23-HBA (the compound from which BBA was synthesized) was reported, where 23-HBA at 2 h attained a C_max_ of 3.1 µg/ml (=6.56 µM) after intragastric administration (IG) of 200 mg/kg 23-HBA [48]. Nevertheless, the potential of BBA to be developed into an adjuvant to chemotherapy still needs to be further investigated, where the clinically effective plasma concentration, drug interaction and pharmacokinetics of BBA in combination with chemotherapeutic agents *in vivo* needs to be studied.

In conclusion, the present study demonstrates that BBA reverses MRP7-mediated MDR by inhibiting the drug efflux function of MRP7. Whether BBA contributes to reversal of clinical MDR mediated by P-gp and/or MRP7 remains to be determined in further study. Altogether, our findings indicate a potentially novel use of BBA as an adjuvant chemotherapeutic agent in clinical practice.

## Supporting Information

Figure S1 **The survival curves of HEK293/pcDNA3.1 and HEK293/MRP7 at different concentrations of BBA.** Cell survival was determined by MTT assay as described in “Materials and Methods”. Data points are the means±SD of triplicate determinations. Experiments were performed at least three independent times.
(TIF)
