# Supplementary figures and images for: BBA, a Synthetic Derivative of 23-hydroxybutulinic Acid, Reverses Multidrug Resistance by Inhibiting the Efflux Activity of MRP7 (ABCC10)
Source: PLoS One. 2013 Sep 17;8(9):e74573. doi: 10.1371/journal.pone.0074573 (PMC3775757; doi:10.1371/journal.pone.0074573)

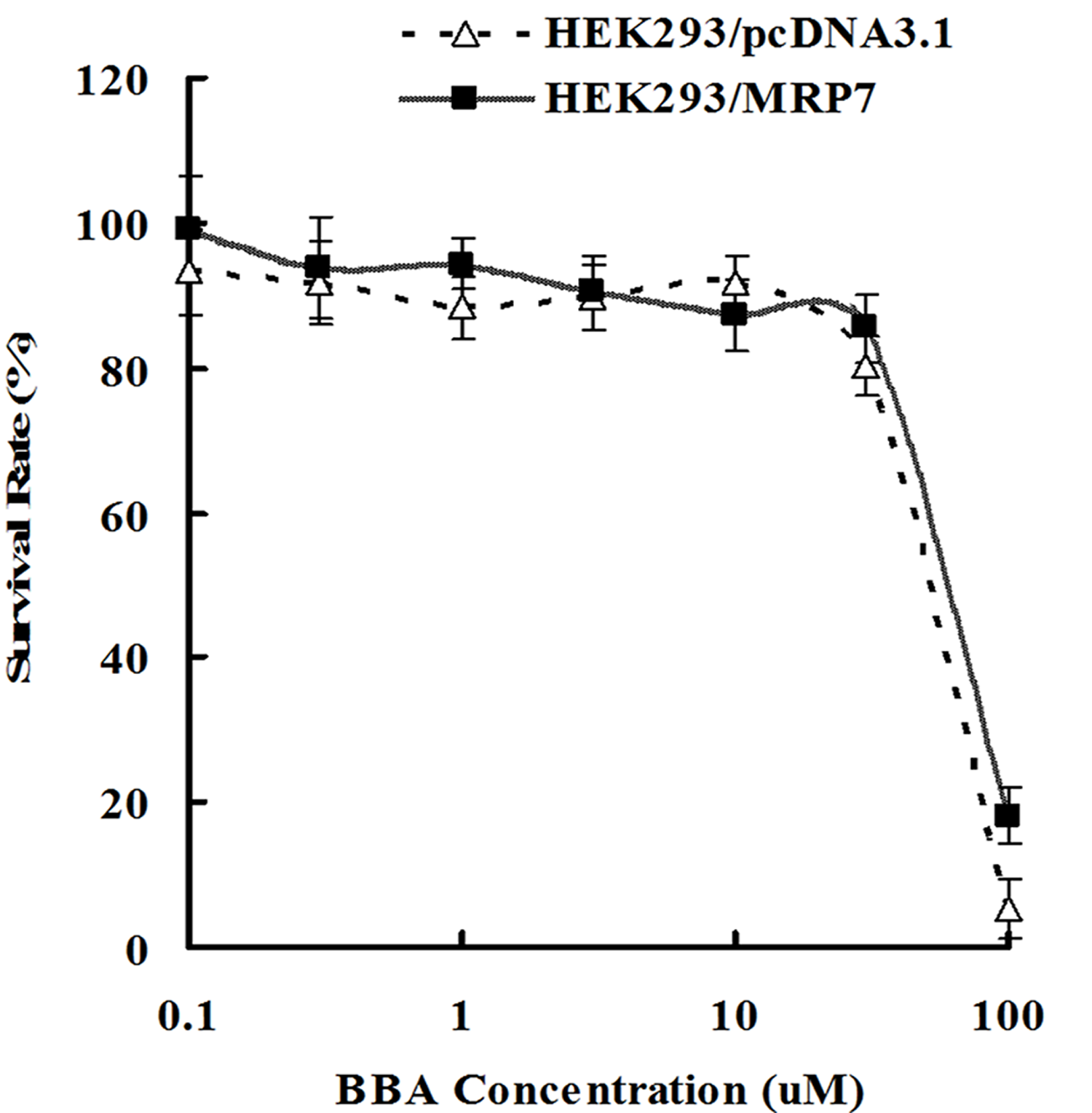

Supplement: Figure S1 — The survival curves of HEK293/pcDNA3.1 and HEK293/MRP7 at different concentrations of BBA. Cell survival was determined by MTT assay as described in “Materials and Methods”. Data points are the means±SD of triplicate determinations. Experiments were performed at least three independent times. (TIF) [file pone.0074573.s001.tif]
